# Clonal hematopoiesis with *DNMT3A* mutation is associated with lower white matter hyperintensity volume

**DOI:** 10.1111/cns.14114

**Published:** 2023-02-21

**Authors:** Woo‐Jin Lee, Keun‐Hwa Jung, Han Song, Heesun Lee, Hyo Eun Park, Youngil Koh, Su‐Yeon Choi, Kyung‐Il Park

**Affiliations:** ^1^ Department of Neurology Seoul National University Bundang Hospital Seongnam‐si South Korea; ^2^ Department of Neurology Seoul National University Hospital Seoul South Korea; ^3^ Genome Opinion Inc. Seoul South Korea; ^4^ Division of Cardiology, Department of Internal Medicine Seoul National University Healthcare System Gangnam Center Seoul South Korea; ^5^ Department of Internal Medicine Seoul National University College of Medicine Seoul South Korea; ^6^ Division of Hemato‐oncology, Department of Internal Medicine Seoul National University Hospital Seoul South Korea; ^7^ Department of Neurology Seoul National University Healthcare System Gangnam Center Seoul South Korea

**Keywords:** clonal hematopoiesis of indeterminate potential, *DNMT3A*, white matter hyperintensity

## Abstract

**Background:**

Clonal hematopoiesis of indeterminate potential (CHIP) increases the risk of cerebrovascular events, while its association with cerebral white matter hyperintensity (WMH) is undemonstrated. We evaluated the effect of CHIP and its major driving mutations on cerebral WMH severity.

**Methods:**

From an institutional cohort of a routine health check‐up program with a DNA repository database, subjects who were ≥50 years of age, with one or more cardiovascular risk factors but no central nervous system disorder, and performed brain MRI were included. Along with the presence of CHIP and its major driving mutations, clinical and laboratory data were obtained. WMH volume was measured in total, periventricular, and subcortical regions.

**Results:**

Among the total 964 subjects, 160 subjects were classified as CHIP positive group. CHIP was most frequently associated with *DNMT3A* mutation (48.8%), followed by *TET2* (11.9%) and *ASXL1* (8.1%) mutations. Linear regression analysis adjusting for age, sex, and conventional cerebrovascular risk factors suggested that CHIP with *DNMT3A* mutation was associated with the lower log‐transformed total WMH volume, unlike other CHIP mutations. When classified according to variant allele fraction (VAF) value of *DNMT3A* mutation, higher VAF classes were associated with the lower log‐transformed total WMH and the lower log‐transformed periventricular WMH volume, but not with the log‐transformed subcortical WMH volumes.

**Conclusions:**

Clonal hematopoiesis with *DNMT3A* mutation is quantitatively associated with a lower volume of cerebral WMH, especially in the periventricular region. CHIP with *DNMT3A* mutation might have a protective role in the endothelial pathomechanism of WMH.

## Graphical abstract

Clonal hematopoiesis with *DNMT3A* mutation is associated with lower white matter hyperintensity volume. In healthy aged population, clonal hematopoiesis of indeterminate potential (CHIP) with *DNMT3A* mutation, the most common driving mutation of CHIP, is associated with lower total WMH volume and WMH volume in the periventricular region. This finding is inconsistent with the previous studies which reported that CHIP increased the risk of major cardiovascular events.

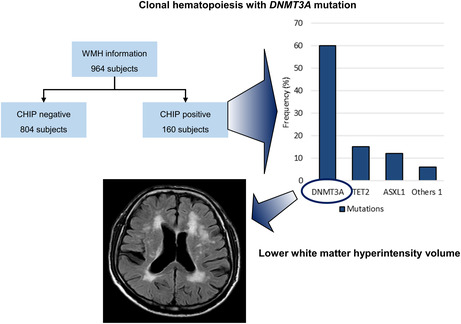

## 1 INTRODUCTION

White‐matter hyperintensity (WMH) is a magnetic resonance image (MRI) marker for cerebral small vessel disease, a highly prevalent aging‐related phenomenon in the brain associated with various neurological complications such as stroke, cognitive dysfunction, and dementia.^1^, ^2^ The fundamental mechanism of WMH pathogenesis has been regarded as the aging‐related reduction of the cerebral small vessel compliance, which induces subsequent endothelial dysfunction, blood–brain barrier disruption, parenchymal hypoperfusion and inflammation, and glymphatic dysfunction.^2^, ^3^, ^4^, ^5^, ^6^, ^7^ However, recent studies indicate that the pathogenesis of WMH might be more complex and factors distinct from the cerebral small vessel compliance could also contribute to the progression of WMH.^8^, ^9^, ^10^, ^11^

Clonal hematopoiesis of indeterminate potential (CHIP) refers to a pre‐malignant status where hematopoietic stem cells with certain somatic mutations expand to form a distinct population of leukocytes in blood circulation (variant allele fraction [VAF] ≥ 2%), but do not satisfy other criteria for diagnosing as a hematologic neoplasm.^12^, ^13^, ^14^ The frequency of CHIP rapidly increases from the age of 60 and reaches up to 10% between the ages of 70 and 79.^12^ In addition to its implication on the risk of hematologic malignancy, the importance of CHIP in the neurological field is rapidly increasing as CHIP increases the risk of major cardiovascular events including ischemic stroke,^12^, ^13^, ^14^, ^15^, ^16^, ^17^, ^18^, ^19^, ^20^, ^21^ while its unexpected protective role on Alzheimer's disease (AD) was also reported.^22^

Given that a small portion of clonal expansion (VAF of 2%) is sufficient to alter systemic atherosclerosis,^12^, ^15^, ^16^ CHIP might have a significant on the pathogenesis of WMH, by affecting the leukocyte‐endothelium interaction during the vascular inflammation process.^23^, ^24^, ^25^ Additionally, the effect of CHIP on WMH pathogenesis might be different according to the major driving mutations of CHIP, which include DNA methyltransferase 3α (*DNMT3A*), tet‐methylcytosine‐dioxygenase 2 (*TET2*), additional‐sex‐combs‐like 1 (*ASXL1*), and Janus kinase 2 (*JAK2*).^12^, ^14^, ^15^, ^18^, ^19^, ^20^, ^21^, ^26^, ^27^, ^28^, ^29^, ^30^ However, the association of CHIP and its driving mutations on cerebral WMH has not been elucidated.

In this study, we investigated the association of CHIP and its major driving mutations on cerebral WMH severity, from a cohort of healthy aged subjects with a DNA repository database.

## 2 METHODS

### 2.1 Study subjects and clinical data

**FIGURE 1:**
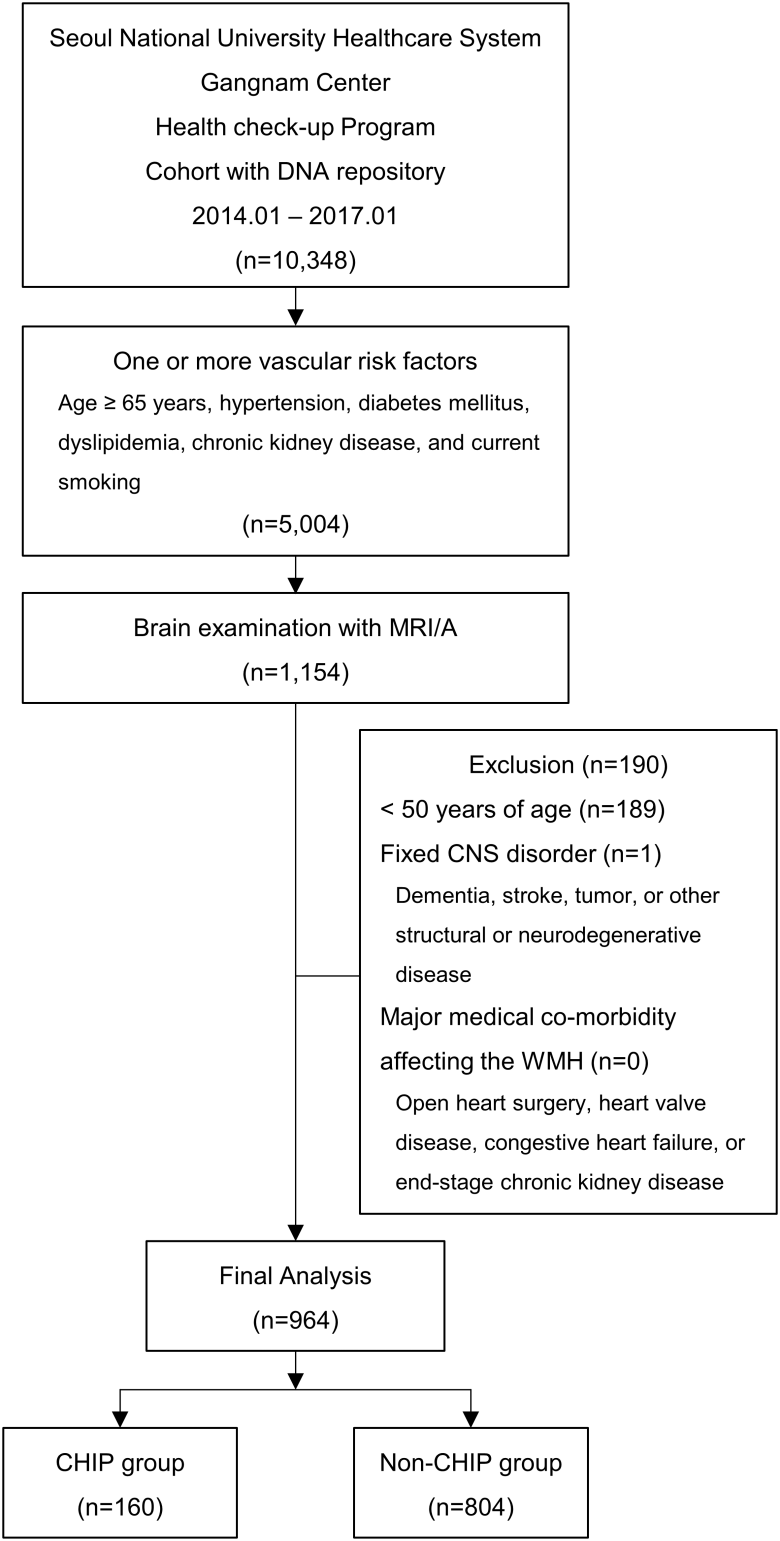
A flow diagram of study participants. CHIP, clonal hematopoiesis of indeterminate potential; CNS, central nervous system; MRI/A, magnetic resonance image/angiography; WMH, white matter hyperintensity.

From an institutional cohort of healthy subjects who voluntarily implemented a routine health check‐up program provided by Seoul National University Healthcare System, Gangnam Center, and were included in the serum DNA repository database by informed consent, all consecutive subjects who were with one or more cardiovascular risk factors (age ≥65 years, hypertension, diabetes mellitus, dyslipidemia, chronic kidney disease, and current smoking) and performed MRI magnetic‐resonance angiography (MRA) within a 1‐year interval between MRI evaluation and acquisition of serum sample were included. Among the initially included 1154 subjects, the final study population was defined according to the following criteria: (1) ≥50 years of age, (2) without a confirmed diagnosis of a central nervous system (CNS) disorder such as dementia, stroke, tumor, or other structural or neurodegenerative disease, (3) without a major cardiac or renal co‐morbidity affecting the WMH status such as open heart surgery, heart valve disease, congestive heart failure, or end‐stage chronic kidney disease.^8^, ^9^, ^31^, ^32^ According to these criteria, 189 subjects with <50 years of age and one subject with a history of territorial stroke were excluded, and the remaining 964 subjects were included in the final analyses (Figure 1). In every subject, the indication for MRI/MRA evaluation was for a regular health check‐up. The Institutional Review Board of the Seoul National University Hospital approved the storage of bio‐specimens with informed consent (IRB no. 1103–127‐357). The bio‐specimens were used retrospectively. The board approved this study protocol (IRB no. H‐1908‐121‐1056), and informed consent for research use of bio‐specimens was waived by the board. The study was conducted in accordance with the Declaration of Helsinki.

Clinical profiles including age, sex, and presence of hypertension, diabetes mellitus, hyperlipidemia, chronic kidney disease, current smoking, ischemic heart disease, and regular use of antithrombotic agents were obtained. Laboratory data were also obtained at the time of DNA sampling, which blood parameters such as hemoglobin (g/dL), hematocrit (%), platelet count and white blood cell count (/μL), glomerular filtration rate (mL/min), hemoglobin A1c (HbA1c, %), total and low‐density lipoprotein (LDL) levels (mg/dL), C reactive protein (mg/dL), and systolic and diastolic blood pressures (mmHg).

### 2.2 Sample preparation, sequencing, and variant calling

After obtaining informed consent for blood storage, blood samples were collected from subjects during a routine health check‐up program. Genomic DNA was separated from peripheral blood and stored at −80°C until sequencing. Targeted sequencing was performed using Agilent SureSelectXT HS custom panel and Illumina NovaSeq 6000 with 2 × 150 bp paired‐end reads and 800× coverage. The custom panel comprises a total exon of 89 genes (*APC, ASXL1, ASXL2, ATM, BCL11B, BCOR, BCORL1, BIRC3, BRAF, BRCC3, CARD11, CASP8, CBL, CD58, CD79B, CHEK2, CNOT3, CREBBP, CUX1, DDX3X, DNMT3A, EP300, ETV6, EZH2, FAM46C, FBXW7, FLT3, FOXP1, GNAS, GNB1, GPS2, HIST1H1C, IDH2, IKZF1, IKZF2, JAK1, JAK2, JAK3, JARID2, KDM6A, KIT, KLHL6, KMT2D, KRAS, LUC7L2, MAP3K1, MPL, MYD88, NF1, NFE2L2, NOTCH1, NOTCH2, NRAS, PDS5B, PDSS2, PHF6, PHIP, PIK3CA, PIK3R1, PPM1D, PRDM1, PRPF40B, PTEN, PTPN11, RAD21, RIT1, RPS15, SETD2, SETDB1, SF1, SF3A1, SF3B1, SMC1A, SMC3, SRSF2, STAG1, STAG2, STAT3, SUZ12, TBL1XR1, TET1, TET2, TNFAIP3, TNFRSF14, TP53, U2AF1, VHL, WT1, ZRSR2*) frequently involved in CHIP. Sequencing reads were trimmed by the quality and aligned to the human genome (hg19) using BWA‐MEM (v0.7.10). Reads were re‐aligned and the base quality score was recalibrated with GATK (v2.3.9). Single nucleotide variants (SNVs) were called using SNver, LoFreq, and GATK. Insertions and deletions were called using an in‐house InDel caller.^15^, ^16^, ^33^, ^34^, ^35^, ^36^ The unreliable variants which did not meet the following criteria were filtered as sequencing artifacts or germline variants: (1) Number of total reads ≥10, number of altered reads at positive strand ≥5 and number of altered reads at negative strand ≥5 with mapping quality value ≥30 and base quality value ≥30; (2) Variant allele frequency falling between 2% and 30%; (3) Does not exist in common germline variants including genomAD, 1k Genome v3, ESP6500, and ExAC; (4) Does not exist in artifact database with minor allele frequency (MAF) > 2% in the internal panel of 1000 Korean individuals.^15^, ^16^, ^33^, ^34^, ^35^, ^36^

### 2.3 Variant annotation

All reliable non‐synonymous variants were annotated as CHIP mutations and the oncogenic variants that have evidence for functional relevance in cancer were annotated as CHIP‐driving mutations according to the following criteria: (1) any truncating mutations including nonsense, splice site mutation, or frameshift indel; (2) any variants previously reported as somatic at least 10 times in solid cancer or at least 1 time with hematopoietic or lymphoid cancer in COSMIC database v83.^15^, ^16^, ^33^, ^34^, ^35^, ^36^

### 2.4 Magnetic resonance imaging analysis

Magnetic resonance image evaluations were performed using 1.5‐T or 3.0‐T units with protocols that included T1‐weighted, T2‐weighted, T2 fluid‐attenuated inversion recovery (FLAIR), gradient echo/susceptibility‐weighted imaging (GRE/SWI), intracranial time‐of‐flight (TOF) MRA, and neck MRA. Parameters for T2‐weighted and FLAIR images were as follows: slice number = 24–30, slice thickness/gap = 4.0–5.0/0.0–2.0 mm, repetition time/echo time = 6000–10,002/92–168.5 milliseconds, field‐of‐view = 285–220 × 285–220 mm, and matrix = 220–512 × 192–400 (see Table S1 for the MRI machine details and parameters).

All MRI images were analyzed by experts blinded to other data. An experienced neurologist (W‐J.L., 10 years of experience) reviewed the MRI to exclude a preexisting ischemic or hemorrhagic stroke lesion, or a significant stenosis in the cerebral arteries. The volumetric analysis of WMH was performed by a neurologist specialist (K‐I. P.), and confirmed by a neurologist (W‐J.L.), according to previously described protocols.^8^, ^9^, ^10^, ^37^, ^38^ Briefly, axial FLAIR images were registered into an offline workstation. Using a freeware NeuRoi (Nottingham University, Nottingham, UK), WMH lesion was identified, the boundaries were designated semi‐automatically, and the volume of each WMH lesion was calculated. The WMH volumes were measured for the total, periventricular, and subcortical regions and normalized by the total cranial volume.^8^, ^9^, ^10^, ^37^, ^38^ The good reproducibility of the volumetric analysis used in this study was established in our previous report.^10^

### 2.5 Statistical analysis

SPSS 25.0 (SPSS Inc.) was used for all statistical analyses. Pearson's *r* was used to evaluate correlations, a two‐tailed *t* test or Mann–Whitney *U* test to compare mean values, and a chi‐square to compare frequencies. Age, sex, conventional vascular risk factors, CHIP or CHIP‐driving mutations of interest, and variables with *p* values <0.10 in univariate analyses were included in regression analyses using enter method. In linear regression analyses, WMH volumes were log‐transformed to obtain a normal distribution (Figure S1). To adjust for a zero value, one‐third of the lowest non‐zero value of WMH volume was uniformly added before log transformation. Unstandardized coefficient B and its 95% confidence interval were used to evaluate the linear association between a parameter and the log‐transformed WMH volume. Variance inflation factor (VIF) was used to check for a multi‐co‐linearity among variables in multiple regression analyses. A scatterplot of the standardized predicted values and the standardized residuals was checked to access the linearity of the regression model. To find a CHIP‐driving mutation with a significant association with WMH volume in a linear regression model, a logistic regression analysis was performed to confirm the association, and the subjects were grouped according to the VAF value, to evaluate its quantitative association with WMH volumes. For all analyses, *p* values <0.05 were considered statistically significant.

## 3 RESULTS

**FIGURE 2:**
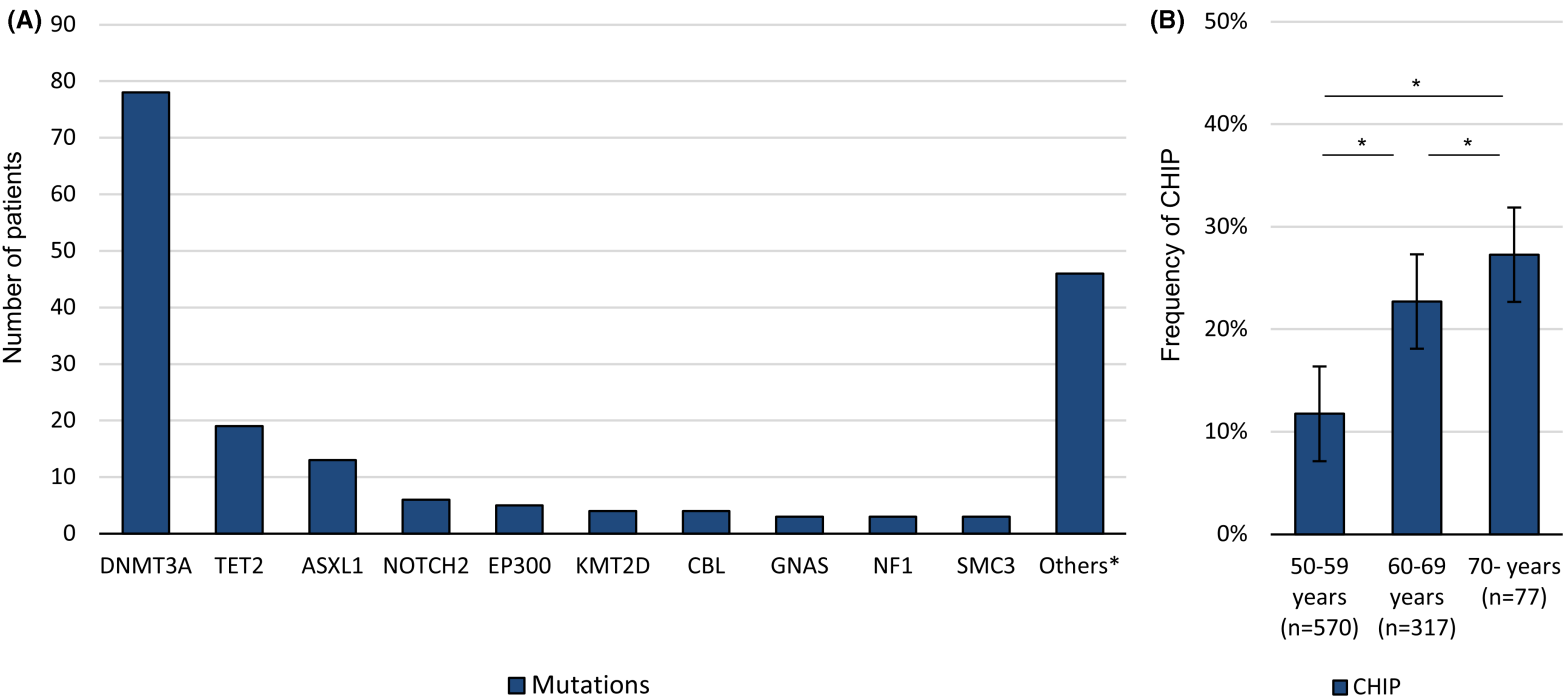
Frequency of Clonal Hematopoiesis of Indeterminate Potential (CHIP) according to age and proportion of driving mutations. Panel A summarizes the frequency of CHIP‐associated mutations with a variant allele fraction of ≥2.0%. Panel B describes the number of subjects and the frequency of CHIP per age group. *one patient each for *SF3A1*, *SUZ12*, *TP53*, *BCL11B*, *CHEK2*, *MAP3K1*, *IKZF2*, *SETD2*, *PRPF40B*, *NOTCH1*, *STAG2*, *APC*, *CUX1*, *BRCC3*, *WT1*, *KIT*, *JAK1*, *PDSS2*, *CD79B*, *MYD88*, *SF3B1*, *KRAS*, *SMC1A*, *GPS2*, and *SF1*, and two subjects each for *GNB1*, *JARID2*, *BCORL1*, *TNFAIP3*, *CREBBP*, *BCOR*, *PPM1D*, *BIRC3*, *ASXL2*, and *TET1*.

Among the total 964 subjects included in this study (237 [24.6%] females, 727 [75.4%] males, mean age 58.9 ± 6.6 years, and range 50–81 years), 160 (16.6%) subjects were classified as CHIP positive (CHIP group) and the remaining 804 (83.4%) as CHIP negative (non‐CHIP group). In the CHIP group, 78 (48.8%) subjects exhibited *DNMT3A* mutation, 19 (11.9%) exhibited *TET2* mutation, 13 (8.1%) exhibited *ASXL1* mutation, and 61 (19.4%) were associated with other mutations (Figure 2A). 24 (15.0%) subjects were positive with two or more mutations. Among the different age groups, a higher age group was associated with a higher frequency of CHIP (*p* < 0.001, Figure 2B). Despite the CHIP group being of higher age compared to the non‐CHIP group (61.4 ± 6.6 years vs. 58.4 ± 6.5 years, *p* < 0.001), cerebrovascular risk factor profiles, laboratory profiles, and WMH severity profiles were comparable between the two groups (Table 1).

**TABLE 1:** Clinical, laboratory, and white matter hyperintensity profiles of the study population.

	Total (*n* = 964)	CHIP (*n* = 160)	Non‐CHIP (*n* = 804)	*p* value
Age (years)	58.9 ± 6.6	61.4 ± 6.6	58.4 ± 6.5	<0.001**
Male sex	727 (75.4%)	115 (71.9%)	612 (76.1%)	0.299
Hypertension (%)	436 (45.2%)	78 (48.8%)	358 (44.5%)	0.372
Diabetes mellitus (%)	173 (17.9%)	33 (20.6%)	140 (17.4%)	0.393
Hyperlipidemia (%)	516 (53.5%)	81 (50.6%)	435 (54.1%)	0.472
Current smoking (%)	178 (18.5%)	22 (13.8%)	156 (19.4%)	0.116
Chronic kidney disease (%)	27 (2.8%)	6 (3.8%)	21 (2.6%)	0.593
Ischemic heart disease (%)	46 (4.8%)	7 (4.4%)	39 (4.9%)	0.956
Regular use of antithrombotic agents (%)	203 (21.1%)	34 (21.2%)	169 (21.0%)	1.000
Laboratory profiles				
Hemoglobin (g/dL)	14.6 ± 1.6	14.6 ± 1.3	14.6 ± 1.7	0.842
Hematocrit (%)	44.2 ± 3.6	44.1 ± 3.8	44.3 ± 3.6	0.556
Platelet (/μL)	226.4 ± 53	228.4 ± 61.3	226.0 ± 51.2	0.469
White blood cell (/μL)	5.5 ± 1.5	5.6 ± 1.7	5.5 ± 1.5	0.939
Glomerular filtration rate (mL/min)	87.1 ± 15.9	84.1 ± 13.7	87.7 ± 16.2	0.555
HbA1c (%)	5.9 ± 0.8	5.9 ± 0.7	5.9 ± 0.8	0.297
Total cholesterol (mg/dL)	161.7 ± 71.4	163.9 ± 68.5	161.2 ± 72.0	0.666
LDL cholesterol (mg/dL)	118.8 ± 34.5	117.8 ± 32.7	119.0 ± 34.8	0.676
C reactive protein (mg/dL)	0.1 ± 0.4	0.1 ± 0.3	0.1 ± 0.4	0.669
Systolic blood pressure (mmHg)	120 ± 13.2	121.6 ± 13.9	119.7 ± 13.1	0.422
Diastolic blood pressure (mmHg)	79.2 ± 9.4	78.9 ± 9.4	79.2 ± 9.4	0.462
WMH volume profiles^a^				
Total volume (%)	0.13 ± 0.22	0.12 ± 0.10	0.13 ± 0.24	0.306
Log‐transformed	−0.99 ± 0.28	−1.00 ± 0.31	−0.99 ± 0.28	
Median [IQR]	−1.00 [−1.14–−0.85]	−1.00 [−1.13–−0.84]	−1.01 [−1.17–−0.87]	
Periventricular volume (%)	0.11 ± 0.09	0.11 ± 0.07	0.11 ± 0.10	0.182
Log‐transformed	−1.03 ± 0.27	−1.06 ± 0.33	−1.02 ± 0.26	
Median [IQR]	−1.02 [−1.16–−0.89]	−1.02 [−1.15–−0.89]	−1.05 [−1.19–−0.91]	
Subcortical volume (%)	0.01 ± 0.03	0.02 ± 0.05	0.01 ± 0.03	0.332
Log‐transformed	−2.42 ± 0.64	−2.40 ± 0.66	−2.43 ± 0.63	
Median [IQR]	−2.59 [−3.00–−1.96]	−2.47 [−3.00–−1.95]	−2.60 [−3.00–−1.96]	

*Note*: Data are reported as a number (percentage), mean ± standard deviation, or median [interquartile range, IQR].

Abbreviations: CHIP, clonal hematopoiesis of indeterminate potential; HbA1c, hemoglobin A1c; LDL, low‐density lipoprotein; WMH, white matter hyperintensity.

^a^ WMH volume was normalized by total cranial volume.

** *p* < 0.01.

In univariate analyses, age and HbA1c were positive, and LDL cholesterol levels were negatively correlated with the log‐transformed total WMH volume (Table 2). The presence of hypertension or diabetes and regular use of antithrombotic agents were associated with a higher log‐transformed total WMH volume, whereas the presence of CHIP or CHIP‐driving mutations was not associated with a significant difference in the log‐transformed total WMH volume (Table 3).

**TABLE 2:** Correlation coefficients of the log‐transformed white matter hyperintensity volume with continuous variables.

	*r*	*p* value
Age (years)	0.296	<0.001**
Hemoglobin (g/dL)	−0.048	0.105
Hematocrit (%)	−0.055	0.071
Platelet (/μL)	−0.047	0.121
White blood cell (/μL)	0.003	0.929
Glomerular filtration rate (mL/min)	−0.007	0.809
HbA1c (%)	0.126	<0.001**
Total cholesterol (mg/dL)	−0.018	0.540
LDL cholesterol (mg/dL)	−0.059	0.047
C reactive protein (mg/dL)	0.012	0.682
Systolic blood pressure (mmHg)	0.032	0.276
Diastolic blood pressure (mmHg)	−0.019	0.517

Abbreviations: HbA1c, hemoglobin A1c; LDL, low‐density lipoprotein; *r*, Pearson's *r* for correlation co‐efficiency; WMH, white matter hyperintensity.

** *p* < 0.01.

**TABLE 3:** Univariate analyses for the factors associated with the log‐transformed white matter hyperintensity volume.

	Log‐transformed Total WMH volume		
	Yes	No	*p* value
Male sex (*n* = 727)	−1.00 ± 0.29	−0.97 ± 0.27	0.235
Hypertension (*n* = 436)	−0.96 ± 0.29	−1.02 ± 0.28	0.002**
Diabetes mellitus (*n* = 173)	−0.95 ± 0.33	−1.00 ± 0.27	0.023*
Hyperlipidemia (*n* = 516)	−1.00 ± 0.27	−0.98 ± 0.3	0.276
Smoking in past 5 years (*n* = 178)	−1.00 ± 0.25	−0.99 ± 0.29	0.556
Chronic kidney disease (*n* = 27)	−0.92 ± 0.38	−0.99 ± 0.28	0.303
Ischemic heart disease (*n* = 46)	−0.94 ± 0.29	−0.99 ± 0.28	0.214
Regular use of antithrombotic agents (*n* = 203)	−0.95 ± 0.32	−1.00 ± 0.27	0.013*
**CHIP**	−0.10 ± 0.03	−0.10 ± 0.03	0.294
CHIP with *DNMT3A* mutation (*n* = 76)	−0.10 ± 0.03	−0.10 ± 0.03	0.239
CHIP with *TET2* mutation (*n* = 18)	−0.09 ± 0.06	−0.10 ± 0.03	0.554
CHIP with *ASXL1* mutation (*n* = 12)	−0.09 ± 0.03	−0.10 ± 0.03	0.222
CHIP with non‐*DNMT3A* mutation (*n* = 84)	−0.10 ± 0.03	−0.10 ± 0.03	0.796

*Note*: Data are reported as mean ± standard deviation. *p < 0.05, **p < 0.01.

Abbreviation: CHIP, clonal hematopoiesis of indeterminate potential.

In the following linear regression analysis, age (B coefficient = 0.001; 95% confidence interval [CI] 0.001–0.001; *p* < 0.001), hypertension (B 0.006; 95% CI 0.002–0.010; *p* = 0.003), and current smoking (B 0.005; 95% CI 0.000–0.010; *p* = 0.033) were associated with higher log‐transformed total WMH volume (Table 4, model 1). When the presence of CHIP was included in the model, the presence of CHIP was significantly associated with the lower log‐transformed total WMH volume, after adjusting age, sex, and conventional cerebrovascular risk factors (B −0.007; 95% CI −0.012–−0.002; *p* = 0.008) (Table 4, model 2), while the model showed better fitness compared to the prior model (*R* ^2^ 0.304 vs. 0.294).

**TABLE 4:** Linear regression analyses for the association of CHIP with log‐transformed total white matter hyperintensity volume.

	B (95% CI)	β	*p* value
Model 1^a^			
Constant variable	−0.173 (−0.209–−0.137)	0.000	<0.001**
Age	0.001 (0.001–0.001)	0.025	<0.001**
Male sex	−0.003 (−0.008–0.002)	−0.004	0.289
Hypertension	0.006 (0.002–0.010)	0.010	0.003*
Diabetes mellitus	0.002 (−0.005–0.008)	0.002	0.620
Hyperlipidemia	0.000 (−0.004–0.004)	0.000	0.899
Chronic kidney disease	0.000 (−0.010–0.011)	0.000	0.931
Current smoking	0.005 (0.000–0.010)	0.008	0.033*
Ischemic heart disease	−0.002 (−0.010–0.007)	−0.001	0.741
Regular use of antithrombotic agents	0.002 (−0.003–0.007)	0.003	0.471
Hematocrit (%)	0.000 (−0.001–0.000)	−0.002	0.585
LDL cholesterol (mg/dL)	0.000 (0.000–0.000)	0.001	0.696
HbA1c (%)	0.003 (−0.001–0.006)	0.007	0.138
Model 2^b^			
Constant variable	−0.183 (−0.211–−0.156)	0.000	<0.001**
CHIP	−0.007 (−0.012–−0.002)	−0.009	0.008**
Age	0.001 (0.001–0.001)	0.026	<0.001**
Male sex	−0.003 (−0.008–0.001)	−0.005	0.151
Hypertension	0.006 (0.002–0.010)	0.010	0.002**
Diabetes mellitus	0.000 (−0.006–0.007)	0.001	0.901
Hyperlipidemia	0.000 (−0.004–0.004)	0.000	0.935
Chronic kidney disease	0.000 (−0.010–0.011)	0.000	0.944
Current smoking	0.005 (0.000–0.010)	0.008	0.031*
Ischemic heart disease	−0.002 (−0.011–0.007)	−0.001	0.694
Regular use of antithrombotic agents	0.002 (−0.004–0.007)	0.002	0.553
Hematocrit (%)	0.000 (0.000–0.000)	−0.002	0.591
LDL cholesterol (mg/dL)	0.000 (0.000–0.000)	−0.001	0.719
HbA1c (%)	0.003 (0.000–0.007)	0.008	0.073
Model 3^c^			
Constant variable	−0.183 (−0.211–−0.156)		<0.001**
CHIP with ***DNMT3A*** mutation	−0.009 (−0.016–−0.002)	−0.009	0.009**
Age	0.001 (0.001–0.001)	0.026	<0.001**
Male sex	−0.003 (−0.008–0.001)	−0.005	0.148
Hypertension	0.006 (0.002–0.010)	0.010	0.002**
Diabetes mellitus	0.000 (−0.006–0.007)	0.001	0.908
Hyperlipidemia	0.000 (−0.004–0.004)	0.000	0.909
Chronic kidney disease	0.000 (−0.010–0.011)	0.000	0.940
Current smoking	0.006 (0.001–0.011)	0.008	0.025
Ischemic heart disease	−0.002 (−0.011–0.007)	−0.001	0.691
Regular use of antithrombotic agents	0.002 (−0.004–0.007)	0.002	0.552
Hematocrit (%)	0.000 (0.000–0.000)	−0.002	0.637
LDL cholesterol (mg/dL)	0.000 (0.000–0.000)	0.000	0.993
HbA1c (%)	0.003 (0.000–0.007)	0.009	0.070

*Note*: Model 1 included demographics, conventional vascular risk factors, and laboratory parameters with *p* values <0.10 in univariate analyses, in the linear regression equation for the factors associated with the log‐transformed total white matter hyperintensity volume. Model 2 additionally included the presence of chip CHIP and Model 3 included the presence of CHIP with ***DNMT3A*** mutation. *p < 0.05, **p < 0.01.

Abbreviations: B, unstandardized coefficient, which represents the slope of the line between the parameter and the log‐transformed WMH volume; HbA1c, hemoglobin A1c; LDL, low‐density lipoprotein; WMH, white matter hyperintensity; β, standardized value of the B coefficient.

^a^ *R* ^2^ = 0.294 and *p* < 0.001 for the linear regression equation.

^b^ *R* ^2^ = 0.304 and *p* < 0.001 for the linear regression equation.

^c^ *R* ^2^ = 0.309 and *p* < 0.001 for the linear regression equation.

Multivariate linear regression analyses were repeated to evaluate the individual effect of major CHIP‐driving mutations on the WMH volume. CHIP with *DNMT3A* mutation was significantly associated with the lower log‐transformed total WMH volume (B −0.009; 95% CI −0.016–−0.002; *p* = 0.009), after adjusting age, sex, and conventional cerebrovascular risk factors (Table 4, model 3). The clinical, laboratory, and cerebrovascular risk factor profiles were similar between the groups with or without CHIP with *DNMT3A* mutation, except for that the group with CHIP with *DNMT3A* mutation was associated with higher age (62.0 ± 6.8 years vs. 58.7 ± 6.5 years, *p* < 0.001) (Table S2). CHIP with *TET2* mutation (B 0.001; 95% CI −0.013–0.014; *p* = 0.929), *ASXL1* mutation (B 0.006; 95% CI −0.010–0.021; *p* = 0.478), or CHIP with non‐*DNMT3A* mutation (B −0.003; 95% CI −0.010–0.003; *p* = 0.315) were not associated with the log‐transformed total WMH volume (Table S3).

To address the issue of small average WMH volume in this study population, subjects were quartalized according to the log‐transformed total WMH volume and logistic regression analyses were performed to confirm the effect of CHIP with *DNMT3A* mutation on WMH volume (Figure S1 for the WMH volume distribution plot). As the result, CHIP with *DNMT3A* mutation exhibited a significant negative association with the highest quartile of log‐transformed WMH volume (Odd ratio = 0.581; 95% CI 0.372–0.907; *p* = 0.017, Table S4).

**FIGURE 3:**
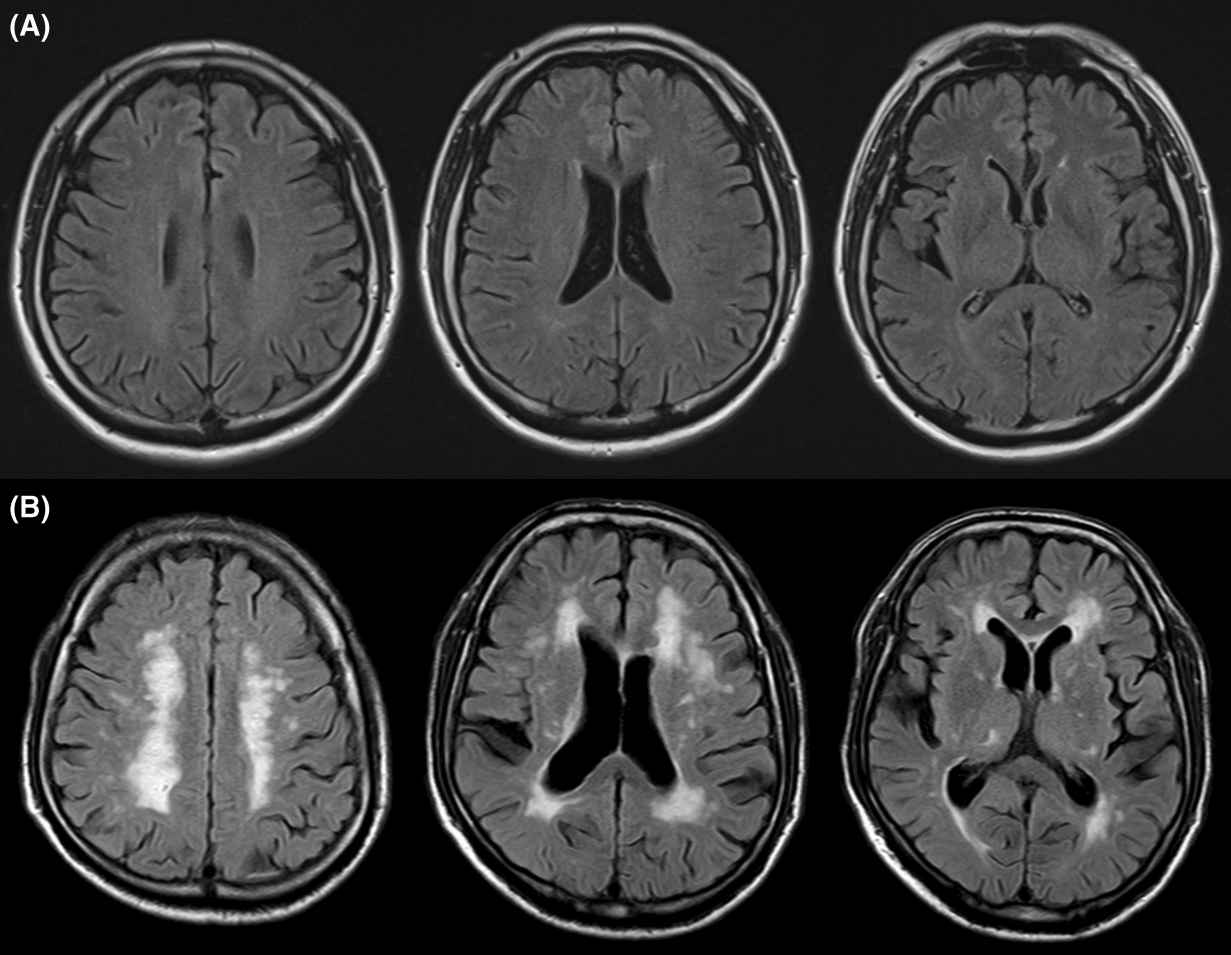
Representative cases. Panel A demonstrates the brain MRI of a 68‐year‐old man with CHIP with *DNMT3A* mutation (variant allele fraction VAF value of 5.8%) and well‐controlled hyperlipidemia (total cholesterol level of 182 mg/dL and LDL cholesterol level of 87 mg/dL) but without other cerebrovascular risk factors. The normalized value of total white matter hyperintensity (WMH) volume was 0.2%. Panel B demonstrates the brain MRI of a 66‐year‐old man with well‐controlled diabetes (hemoglobin A1c of 5.4%) but without CHIP or other cerebrovascular risk factors. The normalized value of total white matter hyperintensity (WMH) volume was 5.9%.

To evaluate the quantitative association of CHIP with *DNMT3A* mutation with the WMH volumes in the total brain and in the brain sub‐regions, subjects were classified according to the VAF value ranges (class 1, non‐ *DNMT3A* mutation, *n* = 886, class 2, CHIP with *DNMT3A* mutation with VAF 2.0–2.9, *n* = 24; class 3, VAF 3.0–4.9, *n* = 29; and class 4, VAF ≥5.0, *n* = 25). Following regression analyses, higher VAF classes of CHIP with *DNMT3A* mutation were associated with the lower log‐transformed total WMH volume (B −0.005 [for one level change in the classes]; 95% CI −0.008–−0.002; *p* = 0.003) and the lower log‐transformed periventricular WMH volume (B −0.004 [for one level change in the classes]; 95% CI −0.008–−0.001; *p* = 0.004), but not with the log‐transformed subcortical WMH volumes (B −0.006 [for one level change in the classes]; 95% CI −0.013–0.001; *p* = 0.082) (Table 5, Figure 3 for the representative images). For all the linear regression analyses, VIF values for the variables were <1.50. In the scatterplot of the standardized predicted values and the standardized residuals, a random and even distribution of the standardized residuals around the zero line was observed.

**TABLE 5:** Linear regression analyses for the association of VAF class of *DNMT3A* mutation with log‐transformed white matter hyperintensity volumes.

	B (95% CI)	β	*p* value
Log‐transformed total WMH volume^a^			
Constant variable	−0.185 (−0.213–−0.157)		<0.001**
VAF class of ***DNMT3A*** mutation^b^	−0.005 (−0.008–−0.002)	−0.010	0.003**
Age	0.001 (0.001–0.001)	0.026	<0.001**
Male sex	−0.003 (−0.008–0.001)	−0.005	0.163
Hypertension	0.006 (0.002–0.010)	0.011	0.002**
Diabetes mellitus	0.000 (−0.007–0.007)	0.000	0.990
Hyperlipidemia	0.000 (−0.004–0.004)	0.000	0.951
Chronic kidney disease	0.001 (−0.010–0.011)	0.000	0.922
Current smoking	0.006 (0.001–0.011)	0.008	0.023
Ischemic heart disease	−0.002 (−0.011–0.007)	−0.001	0.711
Regular use of antithrombotic agents	0.002 (−0.003–0.007)	0.002	0.526
Hematocrit (%)	0.000 (0.000–0.000)	−0.001	0.655
LDL cholesterol (mg/dL)	0.000 (0.000–0.000)	0.000	0.990
HbA1c (%)	0.003 (0.000–0.007)	0.009	0.060
Log‐transformed periventricular WMH volume^c^			
Constant variable	−0.175 (−0.201–−0.148)		<0.001**
VAF class of ***DNMT3A*** mutation^b^	−0.004 (−0.008–−0.001)	−0.010	0.004**
Age	0.001 (0.001–0.001)	0.022	<0.001**
Male sex	−0.003 (−0.007–0.001)	−0.005	0.194
Hypertension	0.004 (0.000–0.007)	0.007	0.054
Diabetes mellitus	0.000 (−0.006–0.007)	0.001	0.891
Hyperlipidemia	0.001 (−0.003–0.005)	0.002	0.611
Chronic kidney disease	−0.003 (−0.013–0.008)	−0.002	0.615
Current smoking	0.005 (0.000–0.010)	0.007	0.054
Ischemic heart disease	0.001 (−0.007–0.010)	0.001	0.751
Regular use of antithrombotic agents	0.001 (−0.004–0.006)	0.001	0.695
Hematocrit (%)	0.000 (0.000–0.000)	0.001	0.670
LDL cholesterol (mg/dL)	0.000 (0.000–0.000)	0.001	0.800
HbA1c (%)	0.003 (−0.001–0.006)	0.008	0.114
Log‐transformed subcortical WMH volume^d^			
Constant variable	−0.436 (−0.489–−0.384)		<0.001**
VAF class of ***DNMT3A*** mutation^b^	−0.006 (−0.013–0.001)	−0.005	0.082
Age	0.003 (0.002–0.003)	0.030	<0.001**
Male sex	−0.007 (−0.017–0.002)	−0.005	0.145
Hypertension	0.017 (0.009–0.025)	0.013	<0.001**
Diabetes mellitus	0.002 (−0.011–0.015)	0.001	0.790
Hyperlipidemia	−0.008 (−0.016–0.000)	−0.006	0.056
Chronic kidney disease	0.031 (0.008–0.055)	0.008	0.010*
Current smoking	0.016 (0.006–0.027)	0.010	0.003**
Ischemic heart disease	−0.010 (−0.029–0.010)	−0.003	0.324
Regular use of antithrombotic agents	0.009 (−0.002–0.02)	0.006	0.106
Hematocrit (%)	0.000 (0.000–0.000)	−0.002	0.540
LDL cholesterol (mg/dL)	0.000 (0.000–0.000)	0.002	0.645
HbA1c (%)	0.003 (−0.003–0.009)	0.004	0.359

*Note*: *p < 0.05, **p < 0.01.

Abbreviations: B, unstandardized coefficient, which represents the slope of the line between the parameter and the log‐transformed WMH volume; HbA1c, hemoglobin A1c; LDL, low‐density lipoprotein; WMH, white matter hyperintensity; β, standardized value of the B coefficient.

^a^ *R* ^2^ = 0.311 and *p* < 0.001 for the linear regression equation.

^b^ VAF class of ***DNMT3A*** mutation was defined according to the VAF value ranges (class 1, no CHIP with *DNMT3A* mutation, *n* = 886; class 2, CHIP with *DNMT3A* mutation with VAF 2.0–2.9, *n* = 24; class 3, VAF 3.0–4.9, *n* = 29; and class 4, VAF ≥5.0, *n* = 25).

^c^ *R* ^2^ = 0.258 and *p* < 0.001 for the linear regression equation.

^d^ *R* ^2^ = 0.360 and *p* < 0.001 for the linear regression equation.

## 4 DISCUSSION

The major finding of this study is that in a healthy aged population, CHIP with *DNMT3A* mutation is associated with lower total WMH volume and WMH volume in the periventricular region, after adjusting age, sex, and conventional cerebrovascular risk factors. Additionally, the subgroups with higher VAF values of CHIP with *DNMT3A* mutation were associated with the lower WMH volume in the total in the periventricular regions.

Although the previous studies which reported the CHIP‐associated increased risk of cardiovascular events^12^, ^16^ this study is the first to demonstrate the specific association of CHIP with *DNMT3A* mutation with lower WMH burden. Considering that CHIP accounts for the significant risk of a major cardiovascular event, the prevalence of CHIP reaches up to 10–20% of the general population >70 years of age and that *DNMT3A* is the most common driving mutation of CHIP, this finding might have a substantial implication on the clinical interpretation and the application of CHIP in the cerebrovascular field.^12^, ^13^, ^14^, ^15^, ^16^

The association of CHIP with *DNMT3A* mutation with lower WMH volume is inconsistent with the findings of the previous studies. In a large‐sized population‐based study, CHIP was associated with 2.0 and 2.6 times higher risk of coronary heart disease and ischemic stroke, respectively.^12^ Another study also reported that CHIP with *DNMT3A* or *TET2* mutations was associated with about two times higher risk of death combined with heart failure hospitalization in chronic heart failure of ischemic origin.^16^ The main mechanism underlying this association was suggested as the infiltrating of clonally expanded circulating leukocytes in the vascular endothelium or myocardium, resulting in activated macrophages that express an increased level of inflammatory molecules such as interleukin (IL)‐1β, IL‐6, IL‐8, C‐X‐C motif ligand (CXCL) 1, CXCL2, and CXCL3, which promotes further recruitment of leukocytes and acceleration of the pathologic processes.^13^, ^14^, ^17^, ^18^, ^19^, ^20^, ^21^

Considering that the proportions of CHIP among the total study population and of *DNMT3A* mutation among the CHIP‐positive population in this study were similar to those in the previous studies (15.6–15.7% and 39.1–40.5, respectively),^12^, ^15^, ^22^, ^36^ the finding of this study might imply that CHIP with *DNMT3A* mutation might have distinct clinical effect from those of other major CHIP‐driving mutations. In a study that included subjects with coronary heart disease and controls, each major CHIP‐driving mutations, *DNMT3A*, *TET2*, *ASXL1*, and *JAK2*, was associated with an increased risk of coronary heart disease, but the hazard ratio was the lowest for the *DNMT3A* mutation.^15^ Remarkably, a recent study reported a potential protective association of CHIP with *DNMT3A* mutation with diabetic polyneuropathy in patients with type 2 diabetes, whereas *TET2* mutation showed a positive association.^36^ They suggested that the conflicting action of *DNMT3A* and *TET2* in DNA methylation and hematopoietic stem cell differentiation might explain their opposite association with diabetic polyneuropathy. While *TET2* is involved in DNA demethylation and myeloid stem cell differentiation, *DNMT3A* mediates de novo methylation of cytosine bases and inhibits hematopoietic stem cell differentiation.^36^ Another study also reported a protective role of CHIP on AD, mediated by the infiltration of mutated myeloid cells into the brain, adopting microglial phenotype, and supplementing the reduced function of endogenous microglia in the aging brain.^22^

Although not demonstrated in this study, distinct biological features of *DNMT3A* might explain the protective effect of CHIP with *DNMT3A* mutation. In WMH pathogenesis, aging‐related decrement of cerebral small vessel compliance has a fundamental role.^4^, ^10^ Unstable and fluctuating shear stress on cerebral microvascular endothelium due to decreased compliance inhibits endothelial synthesis of nitric oxide, aggravates infiltration of inflammatory cells and blood–brain barrier disruption, disturbs parenchymal perfusion and the cerebral waste clearance via the glymphatic system, ultimately resulting in the WMH progression.^2^, ^3^, ^5^, ^6^, ^25^, ^37^, ^39^, ^40^

It is recently suggested that DNMT3A mediates these shear stress‐related processes. DNMT3A expression in vascular endothelium is enhanced by fluctuating shear stress, increases methylation in the promoter of Kruppel‐like factor 4 (KLF4), and inhibits transcription of KLF4.^41^ Decreased KLF4 activity alters the expression of its downward transcription targets, such as decreased nitric oxide synthase 3 and thrombomodulin, and increased monocyte chemoattractant protein‐1, making the vascular environment prone to inflammation and atherosclerosis.^41^, ^42^, ^43^ In this regard, clonally expanded myeloid cells lacking *DNMT3A* with enhanced self‐renewal and cell survival might attenuate this shear stress‐dependent pathologic processes by leukocyte‐endothelium interaction.^23^, ^25^, ^27^, ^28^, ^29^, ^44^

In this study, CHIP with *DNMT3A* mutation was associated with lower WMH volume in the periventricular region. As the periventricular region is supplied by the compliant perforating arterioles directly ramifying from the large cerebral arteries, WMH in the periventricular region is particularly susceptible to the aging‐related reduction in the small vessel compliance.^10^, ^38^, ^45^ Therefore, the protective effect of CHIP with *DNMT3A* mutation on WMH might have been more evident in the periventricular region.

This study has several limitations to be addressed. First, as a cross‐sectional study, the impact of CHIP with *DNMT3A* mutation and its temporal change on the long‐term development or progression of WMH were not examined. Second, due to the small number of subjects with extensive clonal expansion (VAF >10%, *n* = 18, 1.9%), the impact of the extent of clonal expansion on the progression of WMH was not addressed in this study.^16^ Third, the role of clonal expansion with *DNMT3A* mutation on the pathogenesis of WMH was not directly demonstrated by experimental studies. Fourth, due to the low burden of WMH in the study population, the results of this study should not be applied to the relationship between CHIP and major cerebrovascular risk. The lack of animal models that adequately represent the pathomechanism of aging‐related WMH progression might be an obstacle to overcome. Future prospective studies with follow‐up evaluations on the change in the extent of clonal expansion and WMH severity, along with experimental studies with targeted gene editing of the myeloid cells, are warranted to clarify the effect of CHIP with *DNMT3A* mutation on the pathomechanism of cerebral WMH.

In conclusion, clonal hematopoiesis with DNMT3A mutation is quantitatively associated with a lower volume of cerebral WMH, especially in the periventricular region. CHIP with DNMT3A mutation might have a protective role in the endothelial pathomechanism of WMH.

## CONFLICT OF INTEREST STATEMENT

H.S. and Y.K. are shareholders in Genome Opinion, Inc.

## Supporting information


Appendix S1.

## Data Availability

The datasets generated during and/or analyzed during the current study are available from the corresponding author on request.
