# Supplementary material for: Clonal hematopoiesis with DNMT3A mutation is associated with lower white matter hyperintensity volume
Source: CNS Neurosci Ther. 2023 Feb 21;29(5):1243–53. doi: 10.1111/cns.14114 (PMC10068463; doi:10.1111/cns.14114)
Supplement: Supplementary file 1 — Appendix S1. [file CNS-29-1243-s001.docx]

Supplemental Materials

**Mutation specific association of clonal hematopoiesis in determinate potential with white matter hyperintensity**

**List of Supplements: 1 Supplemental Figure and 4 Supplemental Tables**

**Supplemental Figure 1.** Distribution of WMH volume

**Supplemental Table 1.** MRI parameters for the MRI machines included in the analysis

**Supplemental Table 3.** Linear regression analyses for the association of CHIP with other mutations with total white matter hyperintensity volume

**Supplemental Table 4.** Logistic regression analysis for the association of CHIP with *DNMT3A* mutation with the highest quartile of the log-transformed white matter hyperintensity volume

**Supplemental Figure 1.** Distribution of WMH volume


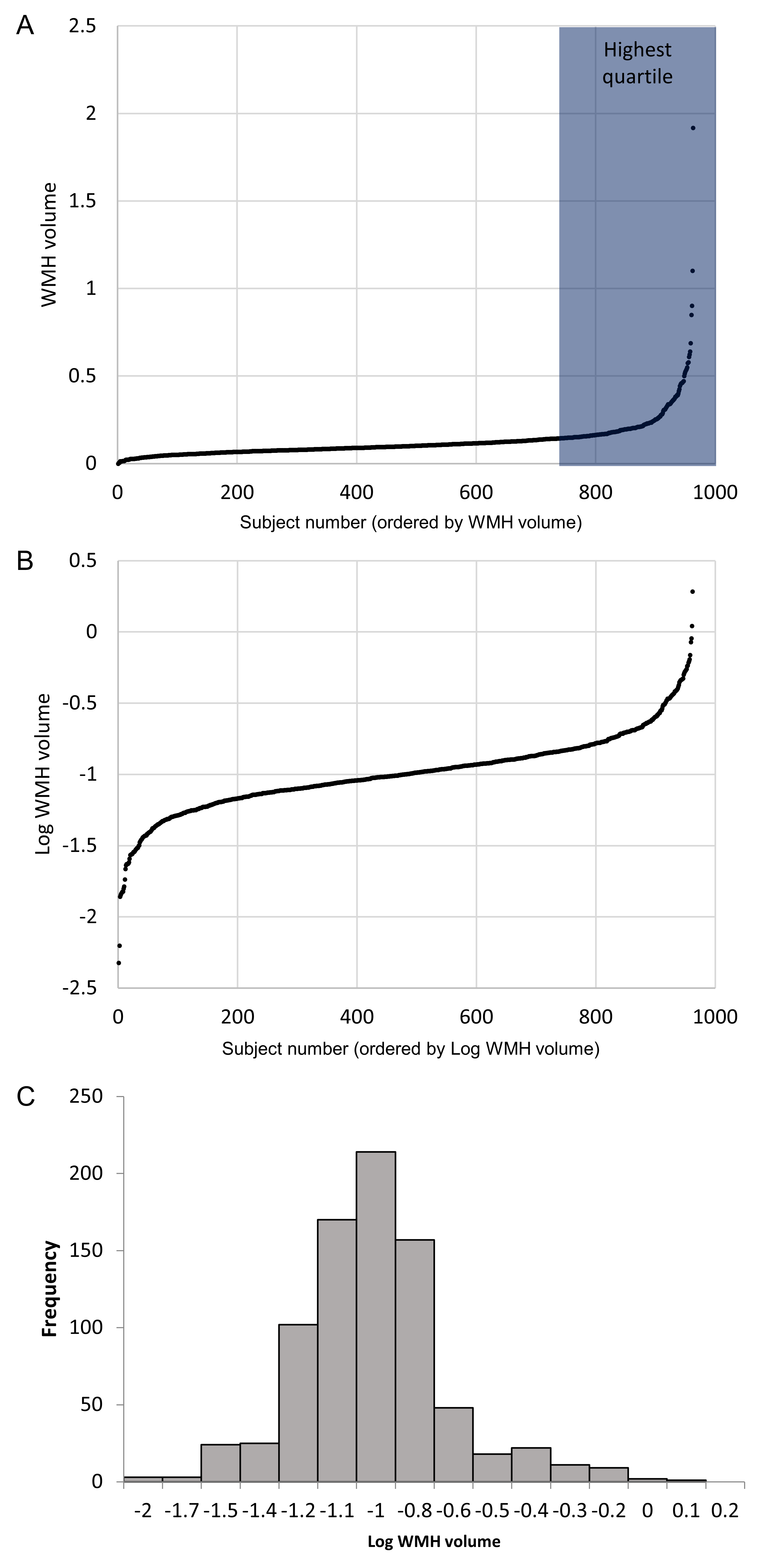


WMH volume (%) showed a highly skewed cumulative distribution pattern (**panel A**). Dark blue rectangle indicates the quartile of the highest WMH volume. Log-transformed WMH volume exhibits a uniform and normal distribution pattern on cumulative distribution graph (**panel B**) and on histogram (**panel C**).

**Supplemental Table 1.** MRI parameters for the MRI machines included in the analysis

|  | GE Signa Excite HD 1.5T | GE Signa Excite 3.0T | GE Genesis Signa 1.5 | GE Discovery MR750w 3.0T | GE Signa Architect 3.0T | Philips Ingenia CX 3.0T | Siemens Skyra 3.0T | Siemens Magnetom vision plus 1.5T | Siemens Magnetom Sonata 1.5T | Siemens Magnetom Trio 3.0T |
| --- | --- | --- | --- | --- | --- | --- | --- | --- | --- | --- |
| FOV  (mm×mm) | 220×220 | 200×200 | 200×200 | 220×220 | 220×220 | 220×220 | 185×220 | 210×210 | 218×220 | 199×200 |
| FA | 90 | 90 | 90 | 142 | 150 | 90 | 150 | 180 | 150 | 140 |
| TR (ms) | 5000 | 9902 | 10002 | 8800 | 9000 | 9000 | 8000 | 9000 | 6000 | 9000 |
| TE (ms) | 108.2 | 168.5 | 123.5 | 89.2 | 92 | 110 | 105 | 119 | 112 | 97 |
| Thickness (mm) | 5 | 5 | 5 | 5 | 5 | 5 | 4 | 5 | 5 | 5 |
| Matrix | 448×256 | 320×192 | 512×140 | 288×288 | 256×256 | 352×225 | 384×204 | 512×126 | 448×400 | 384×209 |
| NEX | 2 | 2 | 1 | 2 | 1 | 1 | 1 | 1 | 2 | 1 |

GE: General electrics, HD: high definition, FOV: field-of-view, FA: flip angle, TR: repetition time, TE: echo time, and NEX: number of excitations.

**Supplemental Table 2.** Comparison of the clinical, laboratory, and white matter hyperintensity profiles between the groups with or without CHIP with *DNMT3A* mutation

|  | **CHIP with *DNMT3A* mutation (n=76)** | **No CHIP with *DNMT3A* (n=888)** | ***P*** |
| --- | --- | --- | --- |
| Age (year) | 62.0±6.8 | 58.7±6.5 | <0.001^**^ |
| Male sex | 53 (69.7%) | 674 (75.9%) | 0.290 |
| Hypertension (%) | 38 (50.0%) | 398 (44.8%) | 0.453 |
| Diabetes mellitus (%) | 14 (18.4%) | 159 (17.9%) | 1.000 |
| Hyperlipidemia (%) | 41 (53.9%) | 475 (53.5%) | 1.000 |
| Current smoking (%) | 13 (17.1%) | 165 (18.6%) | 1.000 |
| Chronic kidney disease (%) | 3 (3.9%) | 24 (2.7%) | 0.788 |
| Ischemic heart disease (%) | 3 (3.9%) | 43 (4.8%) | 0.943 |
| Regular use of antithrombotic agents (%) | 15 (19.7%) | 188 (21.2%) | 0.883 |
| **Laboratory profiles** |  |  |  |
| Hemoglobin (g/dL) | 14.6±1.4 | 14.6±1.6 | 0.972 |
| Hematocrit (%) | 44.1±4.0 | 44.2±3.6 | 0.798 |
| Platelet (/μL) | 222.3±49.9 | 226.8±53.3 | 0.501 |
| White blood cell (/μL) | 5.5±1.5 | 5.5±1.5 | 0.765 |
| Glomerular filtration rate (mL/minute) | 84.3±14.4 | 87.1±15.9 | 0.132 |
| HbA1c (%) | 5.9±0.7 | 5.9±0.8 | 0.465 |
| Total cholesterol (mg/dL) | 159.1±66.5 | 161.9±71.9 | 0.747 |
| LDL cholesterol (mg/dL) | 115.9±28.3 | 119.1±35.0 | 0.357 |
| C reactive protein (mg/dL) | 0.1±0.1 | 0.1±0.4 | 0.202 |
| Systolic blood pressure (mmHg) | 121.4±15.2 | 119.9±13.0 | 0.329 |
| Diastolic blood pressure (mmHg) | 79.3±9.3 | 79.1±9.4 | 0.864 |
| **WMH volume profiles**^†^ |  |  |  |
| Total volume (%) | 0.12±0.09 | 0.13±0.23 | 0.281 |
| Log-transformed | -1.03±0.30 | -0.99±0.28 |  |
| Median [IQR] | -1.05 [-1.19‒-0.88] | -1.00 [-1.13‒-0.85] |  |
| Periventricular volume (%) | 0.10±0.07 | 0.11±0.09 | 0.182 |
| Log-transformed | -1.07±0.30 | -1.02±0.27 |  |
| Median [IQR] | -1.08 [-1.22‒-0.90] | -1.02 [-1.15‒-0.89] |  |
| Subcortical volume (%) | 0.01±0.04 | 0.01±0.03 | 0.332 |
| Log-transformed | -2.37±0.66 | -2.43±0.64 |  |
| Median [IQR] | -2.47 [-3.00‒-1.84] | -2.59 [-3.00‒-1.96] |  |

Data are reported as a number (percentage), mean±standard deviation, or median [interquartile range, IQR]. CHIP, clonal hematopoiesis of indeterminate potential, HbA1c, hemoglobin A1c, LDL, low-density lipoprotein, and WMH, white matter hyperintensity. ^†^WMH volume was normalized by total cranial volume. ^**^*P*<0.01.

**Supplemental Table 3.** Linear regression analyses for the association of CHIP with other mutations with log-transformed total white matter hyperintensity volume

| **Log-transformed Total WMH volume^a^** | **B (95% CI)** | **β** | ***P*** |
| --- | --- | --- | --- |
| Constant variable | -0.181 (-0.208–-0.153) |  | <0.001^**^ |
| CHIP with ***TET2*** mutation | 0.001 (-0.013–0.014) | 0.000 | 0.929 |
| Age | 0.001 (0.001–0.001) | 0.024 | <0.001^**^ |
| Male sex | -0.003 (-0.008–0.001) | -0.005 | 0.179 |
| Hypertension | 0.006 (0.002–0.010) | 0.010 | 0.003^**^ |
| Diabetes mellitus | 0.000 (-0.006–0.007) | 0.000 | 0.927 |
| Hyperlipidemia | 0.000 (-0.004–0.004) | 0.000 | 0.936 |
| Chronic kidney disease | 0.000 (-0.01–0.011) | 0.000 | 0.952 |
| Smoking in past 5 years | 0.005 (0.000–0.010) | 0.007 | 0.033^*^ |
| Ischemic heart disease | -0.002 (-0.011–0.007) | -0.001 | 0.716 |
| Regular use of antithrombotic agents | 0.002 (-0.003–0.007) | 0.002 | 0.513 |
| Hematocrit (%) | 0.000 (0.000–0.000) | -0.002 | 0.507 |
| LDL cholesterol (mg/dL) | 0.000 (0.000–0.000) | 0.000 | 0.976 |
| HbA1c (%) | 0.003 (0.000–0.007) | 0.009 | 0.064 |
| **Log-transformed Total WMH volume^b^** | **B (95% CI)** | **β** | ***P*** |
| Constant variable | -0.18 (-0.208–-0.153) |  | <0.001^**^ |
| CHIP with ***ASXL1*** mutation | 0.006 (-0.010–0.021) | 0.002 | 0.478 |
| Age | 0.001 (0.001–0.001) | 0.024 | <0.001^**^ |
| Male sex | -0.003 (-0.008–0.001) | -0.005 | 0.185 |
| Hypertension | 0.006 (0.002–0.010) | 0.010 | 0.003^**^ |
| Diabetes mellitus | 0.000 (-0.006–0.007) | 0.000 | 0.931 |
| Hyperlipidemia | 0.000 (-0.004–0.004) | 0.000 | 0.985 |
| Chronic kidney disease | 0.000 (-0.011–0.011) | 0.000 | 0.964 |
| Smoking in past 5 years | 0.005 (0.000–0.010) | 0.007 | 0.038 |
| Ischemic heart disease | -0.002 (-0.011–0.007) | -0.001 | 0.729 |
| Regular use of antithrombotic agents | 0.002 (-0.003–0.007) | 0.003 | 0.504 |
| Hematocrit (%) | 0.000 (0.000–0.000) | -0.002 | 0.518 |
| LDL cholesterol (mg/dL) | 0.000 (0.000–0.000) | 0.000 | 0.974 |
| HbA1c (%) | 0.003 (0.000–0.007) | 0.009 | 0.060 |
| **Log-transformed Total WMH volume^c^** | **B (95% CI)** | **β** | ***P*** |
| Constant variable | -0.181 (-0.209–-0.153) |  | <0.001^**^ |
| CHIP with **non-*DNMT3A*** mutation | -0.003 (-0.010–0.003) | -0.003 | 0.315 |
| Age | 0.001 (0.001–0.001) | 0.025 | <0.001^**^ |
| Male sex | -0.003 (-0.008–0.001) | -0.005 | 0.178 |
| Hypertension | 0.006 (0.002–0.010) | 0.010 | 0.003^**^ |
| Diabetes mellitus | 0.000 (-0.006–0.007) | 0.000 | 0.924 |
| Hyperlipidemia | 0.000 (-0.004–0.004) | 0.000 | 0.940 |
| Chronic kidney disease | 0.000 (-0.010–0.011) | 0.000 | 0.952 |
| Current smoking | 0.005 (0.000–0.010) | 0.007 | 0.036 |
| Ischemic heart disease | -0.002 (-0.011–0.007) | -0.001 | 0.708 |
| Regular use of antithrombotic agents | 0.002 (-0.003–0.007) | 0.002 | 0.511 |
| Hematocrit (%) | 0.000 (0.000–0.000) | -0.002 | 0.505 |
| LDL cholesterol (mg/dL) | 0.000 (0.000–0.000) | 0.000 | 0.966 |
| HbA1c (%) | 0.003 (0.000–0.007) | 0.009 | 0.066 |

^a^R^2^=0.297 and *P*<0.001 for the linear regression equation.

^b^R^2^=0.296 and *P*<0.001 for the linear regression equation.

^c^R^2^=0.298 and *P*<0.001 for the linear regression equation.

B, unstandardized coefficient, β, standardized coefficient, WMH, white matter hyperintensity, HbA1c, hemoglobin A1c, and LDL, low-density lipoprotein. ^*^*P*<0.05, ^**^*P*<0.01.

**Supplemental Table 4.** Logistic regression analysis for the association of CHIP with *DNMT3A* mutation with the highest quartile of the log-transformed white matter hyperintensity volume

| **Highest quartile of WMH volume** | **Odd Ratio (95% CI)** | ***P*** |
| --- | --- | --- |
| CHIP with ***DNMT3A*** mutation | 0.581 (0.372–0.907) | 0.017^*^ |
| Age | 1.095 (1.067–1.124) | <0.001^**^ |
| Male sex | 0.765 (0.522–1.121) | 0.169 |
| Hypertension | 2.025 (1.465–2.800) | <0.001^**^ |
| Diabetes mellitus | 1.133 (0.648–1.980) | 0.661 |
| Hyperlipidemia | 0.912 (0.661–1.258) | 0.573 |
| Chronic kidney disease | 1.295 (0.531–3.154) | 0.570 |
| Current smoking | 1.487 (0.959–2.305) | 0.076 |
| Ischemic heart disease | 1.243 (0.592–2.611) | 0.565 |
| Regular use of antithrombotic agents | 0.708 (0.459–1.094) | 0.120 |
| Hematocrit (%) | 0.995 (0.983–1.006) | 0.365 |
| LDL cholesterol (mg/dL) | 1.000 (0.995–1.005) | 0.949 |
| HbA1c (%) | 1.107 (0.827–1.480) | 0.495 |

R^2^=0.158 and *P*<0.001 for the logistic regression equation.

WMH, white matter hyperintensity, HbA1c, hemoglobin A1c, and LDL, low-density lipoprotein. ^*^*P*<0.05, ^**^*P*<0.01.
